# Lhx2 Regulates Distinct Dendritic Architecture of Pyramidal Neurons across Primary Sensory Areas

**DOI:** 10.1523/ENEURO.0103-26.2026

**Published:** 2026-08-25

**Authors:** Achintya Srivastava, Deyl S. Djama, Dipanjana Banerjee, Shubha Tole, Leena Ali Ibrahim

**Affiliations:** ^1^Department of Biological Sciences, Tata Institute of Fundamental Research, Mumbai 400005, India; ^2^Biomedical Sciences Division, King Abdullah University of Science and Technology, Thuwal 23955-6900, Saudi Arabia

**Keywords:** dendritic arbors, excitability, LHX2, pyramidal neurons, somatosensory cortex, visual cortex

## Abstract

Distinct neocortical regions subserve different sensory functions, yet the cellular features that distinguish neurons across cortical areas remain poorly understood. Layer (L)2/3 pyramidal neurons (PyNs) are generated at similar developmental times throughout the cortex, but whether their morphological and functional properties are shaped by areal identity programs is unclear. Here, we compared L2/3 PyNs in mouse primary somatosensory (S1) and primary visual (V1) cortices (both male and female). We observed pronounced areal differences where V1 neurons exhibited smaller and less complex dendritic arbors and displayed increased intrinsic excitability relative to their counterparts in S1. These differences were present in both juvenile and adult stages, indicating that they emerge early and persist over time. Given prior evidence implicating the transcription factor LHX2 in dendritic arborization, we tested its contribution to these differences. Loss of *Lhx2* in E15.5 V1 progenitors did not alter neuronal morphology in V1, in contrast to our previous findings in S1. We found that E15.5 progenitors display lower levels of LHX2 protein in V1 than in S1; therefore, we overexpressed *Lhx2* in V1 progenitors and found increased dendritic branching complexity in V1 L2/3 PyNs. Together, these findings identify LHX2 as a molecular regulator that contributes to area-specific structural differentiation of L2/3 PyNs. Our results show that PyNs arising from different regions of the dorsal pallium diverge in morphology and physiology according to the cortical area, suggesting that regionally patterned transcriptional programs help establish functional specialization across the neocortex.

## Significance Statement

What mechanisms give rise to the vast diversity of neuronal features required for sensory processing? Our findings demonstrate that pyramidal neurons acquire area-specific morphological and physiological fingerprints that are established early and persist through adulthood. We identify LHX2 as a key regulator of mature neuronal identity, providing a framework for understanding how regional specialization is hardwired into the neocortical architecture.

## Introduction

The mammalian neocortex is subdivided into functionally specialized areas. Anatomical and molecular evidence suggest that excitatory neurons are not uniform across the cortex but display distinct morphological, molecular, and connectivity features (Harris and Shepherd, 2015). Comparative studies have shown that pyramidal neuron (PyN) dendritic architecture varies systematically between cortical areas across different species (Benavides-Piccione et al., 2006; Reyes et al., 2016; González-Burgos et al., 2019). More recently, large-scale single-cell transcriptomic analyses revealed substantial molecular divergence among excitatory neurons across cortical regions even in similar layers, whereas inhibitory neuron classes appear comparatively conserved (Tasic et al., 2018; Yao et al., 2021). These findings suggest that PyNs may acquire area-specific identities, yet direct comparisons linking area-specific regulatory mechanisms to differences in neuronal morphology and intrinsic physiology remain limited.

Layer (L)2/3 PyNs constitute a major class of intracortical projection neurons and provide much of the callosal connectivity linking the two hemispheres, thereby supporting integration of sensory information across cortical areas. These neurons receive modality-specific input, either directly from thalamic afferents or through local cortical circuits, depending on the sensory region in which they reside. This raises the question of how area-specific features are acquired by neurons in distinct cortical regions.

To address this, we focused on two primary sensory areas, the somatosensory (S1) and the primary visual cortex (V1), and directly compared the morphological and intrinsic physiological properties of their L2/3 PyNs. We found that L2/3 PyNs in the adult differ systematically between S1 and V1 in dendritic architecture and intrinsic excitability and that these distinctions are already evident at juvenile stages. In mice, L2/3 neurons are generated around embryonic day (E)15.5 (Angevine and Sidman, 1961; Anderson and Vanderhaeghen, 2014). In prior work, we found that LIM-HD transcription factor LHX2 regulates the dendritic morphogenesis and neuronal properties of S1 L2/3 PyNs (Bose et al., 2025). Loss of *Lhx2* from E15.5 progenitors resulted in L2/3 PyNs with shrunken morphologies and altered electrophysiological properties (Bose et al., 2025). Here, we examine whether this mechanism operates in V1 as well. Surprisingly, we find that LHX2 levels were lower in V1 compared with S1 progenitors, and loss of *Lhx2* does not alter neuronal morphology in V1. Furthermore, overexpression of *Lhx2* in V1 progenitors caused a small but significant increase in the dendritic branching complexity of the resulting postmitotic L2/3 PyNs. These findings identify an area-specific mechanism that shapes dendritic arborization of L2/3 PyNs.

## Materials and Methods

### Animals

Mice were bred and housed at King Abdullah University of Science and Technology, in accordance with the guidelines of the local Institutional Animal Care and Use Committee protocol number LI23IACUC003, and at Tata Institute of Fundamental Research (TIFR; TIFR-IAEC). Patch-clamp experiments were conducted using juvenile *Lhx2^lox/lox^::Ai9/Ai9* (P28–P35) and adult (∼8 weeks old) C57BL/6 mice of both sexes.

Inutero electroporation (IUE) experiments regarding generation of Lhx2 knock-out neurons were performed in *Lhx2^lox/lox^* mice. *Lhx2* overexpression experiments were performed on Swiss mice.

Mice were housed in a standard 12 h light/dark cycle and allowed *ad libitum* access to food and water. Noon of the day of the vaginal plug was designated as E0.5. Both male and female animals were used for all experiments.

### Genotyping

Primers for genotyping were as follows (expected band sizes):

*Lhx2* conditional forward, ACCGGTGGAGGAAGACTTTT; *Lhx2* conditional reverse, CAGCGGTTAAGTATTGGGACA (WT, 144 bp; *Lhx2lox*, 188 bp).

Cre Forward primer sequence, ATTTGCCTGCATTACCGGTC; Cre Reverse primer sequence, ATCAACGTTTTCTTTTCGG (Cre band, 350 bp).

### In-utero electroporation (IUE)

IUE was performed at E15.5, as previously described (Pal et al., 2021). Embryos were injected with plasmid DNA solution dissolved in nuclease-free water with 0.1% fast green with plasmid DNA into the lateral ventricle through the uterine wall using a fine glass microcapillary (Sutter capillaries number B100-75-10).

The pCAGGS-IRES-tdTomato construct was obtained from Addgene (number 83029). pCAGGS-IRES-Cre-tdTomato was generated in the lab.

Membrane-bound EGFP (CAAX-EGFP) was a gift from M. Sonawane (TIFR, India).

Lhx2-tdTomato plasmid used was a dual-promoter vector. pCRC, which expresses tdTomato, downstream of the first CAG promoter were used (gift from Toshio Ohshima, Waseda University, Tokyo, Japan). A 1.2-kb EcoRI digest of pcDNA3.1- Lhx2 (gift from Leif Carlsson, Umeå Centre for Molecular Medicine, Umeå, Sweden) containing the *Lhx2* ORF was ligated into the EcoR1 site downstream of the second CAG promoter.

### Histology

Brains from mice electroporated at E15.5 were harvested at postnatal day (P)30–P34. Mice were first anesthetized using thiopentone and transcardially perfused with saline, followed by 4% (w/v) paraformaldehyde in phosphate buffer (PB). Brains were kept for overnight fixation and then cryoprotected by transferring to 30% sucrose in PBS until sectioning. The brains were sectioned at 50 μm using a microtome (Leica SM201R).

For E15.5 brains, pregnant mice were killed at 15.5 DPC; the uterine horn was dissected out to obtain E15.5 embryos. The embryonic E15.5 brains were transferred to vials with 4% (w/v) paraformaldehyde in PB and kept on a rocker for 4 h at room temperature and then transferred to 30% sucrose–phosphate-buffered saline at 4°C until sectioning. The brains were sectioned at 25 μm using a microtome.

### Immunohistochemistry

Brains were sectioned at 25 or 50 μm and processed for free-floating immunohistochemistry. Sections were washed and permeabilized with PB containing 0.3% (v/v) Triton X-100. Blocking was done with 5% (v/v) horse serum in PB with 0.3% (v/v) Triton X-100 for 1 h at room temperature. Primary antibody treatment was performed in PB containing 0.3% (v/v) Triton X-100 with 2.5% (v/v) horse serum and incubated overnight at 4°C. The sections were washed in PB, followed by the appropriate secondary antibody for 2 h at room temperature and 4′,6-diamidino-2-phenylindole. Sections were mounted on SuperFrost Plus glass microscope slides (catalog #71869-10) and protected with Fluoroshield (Sigma-Aldrich catalog #F6057 or F6182). Antibodies used were as follows: biotinylated GFP (Abcam; ab6658, 1:200) and rabbit red fluorescent protein (Abcam; ab65856, 1:200). For quantification of LHX2 levels (Fig. 3*A*), coronal sections from three independent brains were processed together in a single well of a four-well plate. After immunostaining, they were mounted on a single slide and imaged using the same parameters for S1 and V1.

Slices with biocytin-filled neurons were fixed in 4% paraformaldehyde and stored in the fridge at 4°C overnight. Slices were first washed with PB, then incubated with 0.5% (v/v) Triton X-100 in PB for 30 min at RT, and then incubated with Streptavidin Alexa Fluor 488 (Invitrogen; S11223, 1:200) in 0.5% (v/v) Triton X-100 in PB for 48 h. Slices were washed again and mounted with DAPI Fluoromount-G (catalog #00-4959-52).

### Image acquisition

Images of stained electroporated neurons were obtained on Olympus FluoView 1200 and 3000 confocal microscopes with the FluoView software using a 40× objective.

Images of Lhx2 overexpressing L2/3 PyNs and WT controls were taken on the Leica Sp8 confocal system. Biocytin-filled neurons from patch-clamp recording were imaged on Leica SP8 confocal microscope using a 40× objective. All confocal images of neurons had a 0.8 μm stack. All images were acquired at 1,024 × 1,024 pixel resolution. For imaging of neurons used for reconstruction, laser power and gain were adjusted such that the dendrites are clearly visible and discernible from the background, with each neuron imaged at its optimum acquisition setting. For imaging progenitors for LHX2 levels, laser power, gain, PMT voltage, and offset were set using the first image to establish a dynamic range (with both oversaturated and zero signal pixels present on the same image), and these settings were then applied to all subsequent images.

### Morphological reconstruction of neuronal dendrites

A 3D morphology reconstruction of CAAX-EGFP labeled and biocytin-filled neurons was done using the Neurolucida 2017 software and Neurolucida 360, respectively. The apical and basal dendrites were identified based on the directionality of the dendrites. The primary dendrite directed toward the pial surface of the cortex was considered apical, and the rest were labeled as basal. Total length, mean length, and Sholl analysis were performed using the Neurolucida Explorer 2017 software. For calculating the normalized cortical depth, the distance of the neuronal soma from the pial surface was normalized with cortical thickness (distance from the pial surface to the lower boundary of L6).

### Whole-cell patch–clamp electrophysiology

#### Brain slice preparation

Mice were killed by cervical dislocation; the brain was quickly dissected and put in an ice-cold slicing solution constantly bubbled with 95% O_2_ and 5% CO_2_. The slicing solution, pH 7.3 (∼300–310 mOsm), solution contained the following (in mM): 210 sucrose, 2.8 KCl, 2 MgSO_4_, 1.25 Na_2_HPO_4_, 26 NaHCO_3_, 6 MgCl_2_, 1 CaCl_2_, and 10 d-glucose saturated with carbogen (95% O_2_ and 5% CO_2_). Thereafter 300-μm-thick coronal slices were prepared with a vibrating blade microtome (Leica VT1000S vibratome) while submerged in an ice-cold slicing solution with slicing solution ice slurry and saturated with carbogen. The slices were then incubated for 30 min at 34°C in a holding chamber containing a holding solution, pH 7.3 (∼300–310 mOsm), with the composition of the following (in mM): 125 NaCl, 2.5 KCl, 1.25 NaH_2_PO_4_, 25 NaHCO_3_, 1 CaCl_2_, 2 MgCl_2_, and 10 d-glucose saturated with carbogen. Thereafter, the slices were kept in the holding chamber at room temperature for at least 1 h before recordings started.

#### Whole-cell current–clamp recordings

For electrophysiological recordings, slices were transferred to the recording chamber and were continuously perfused with carbogenated artificial cerebrospinal fluid (ACSF; extracellular recording solution) at a flow rate of 2–3 ml/min. All neuronal recordings were performed under current-clamp configuration at room temperature. The ACSF contained the following (in mM): 125 NaCl, 2.5 KCl, 1.25 NaH_2_PO_4_, 25 NaHCO_3_, 2 CaCl_2_, 2 MgCl_2_, and 10 d-glucose, pH 7.3 (∼300–310 mOsm). Slices were first visualized under a 4× objective lens to visually locate L2/3 of the somatosensory barrel field (S1) or V1 of the visual cortex (Orca-Flash, Hamamatsu). A 40× water-immersion objective lens was used to perform visually guided patch-clamp recordings of L2/3 PyNs (Scientifica; Olympus, U-TV1XC). Whole-cell current–clamp recordings were performed from L2/3 PyNs using a Multiclamp 700B amplifier and digitized using Digidata 1550B digitizer (Molecular Devices) with a sampling rate of 25 kHz and Bessel filter of 10 kHz.

Borosilicate glass electrodes with electrode tip resistance between 3 and 7 MΩ were pulled (P-1000 micropipette puller; Sutter) from thick glass capillaries (GC150F-10; Harvard Apparatus) and used for patch-clamp recordings. The pipette solution contained the following (in mM): 120 K-gluconate, 20 KCl, 10 HEPES, 4 NaCl, 4 Mg–adenosine 5′-triphosphate, and 0.3 Na–guanosine 5′-triphosphate, pH 7.3 (adjusted with KOH; osmolarity ∼300 mOsm). Series resistance was monitored and compensated online with the bridge-balance circuit of the amplifier. Voltages have not been corrected for the liquid junction potential, which was experimentally measured to be ∼10 mV. Capacitance was not compensated for the pipette in current-clamp mode.

*Exclusion criteria:* Cells were excluded if the initial resting membrane potential (RMP) was more depolarized than −60 mV, if series resistance rose above 30 MΩ, and if series resistance showed a >30% change from its initial value.

#### Voltage-clamp recordings

In the whole-cell patch–clamp mode, with internal solution consisting of (in mM) 20 K-gluconate, 20 KCl, 10 HEPES, 4 NaCl, 4 Mg–adenosine 5′-triphosphate, and 0.3 Na–guanosine 5′-triphosphate, pH 7.3 (adjusted with KOH; osmolarity ∼300 mOsm), the amplifier was switched to voltage-clamp mode, and the cell was held at −70 mV. Spontaneous excitatory synaptic events were recorded for 3–5 min using a Gap-free protocol. Recordings were sampled at 50 kHz filtered at 1 kHz with a gain value of 10. Slow and fast capacitance was compensated in voltage-clamp mode.

#### Analyses of electrophysiological data

Whole-cell current–clamp recordings were performed using a series of current injections ranging from −200 to 320 pA in 20 pA increments, with each step lasting 1 s and preceded by a −50 pA prepulse. For rheobase measurements, a separate protocol was used in which depolarizing current steps of 10 pA increments were applied starting from 0 pA until the first action potential was elicited.

*Subthreshold membrane properties* were derived from voltage responses to hyperpolarizing current injections. RMP was defined as the baseline membrane voltage in the absence of current injection. Input resistance (IR) was calculated both from the steady-state voltage deflection at −200 pA divided by −200 pA and from the slope of the current–voltage (*I*–*V*) relationship using subthreshold responses up to the last nonspiking current step. Sag ratio was defined as the ratio between the peak and steady-state voltage deflections at −200 pA. The membrane time constant (*τ*) was obtained by fitting the exponential decay of the voltage response to the −50 pA prepulse, and membrane capacitance (*C*) was calculated as *C* = *τ*/IR.

*Suprathreshold firing properties* were extracted from depolarizing current injections. Firing frequency was defined as the number of action potentials generated during each current step. Rheobase was determined as the minimum current required to evoke the first action potential using 10 pA incremental steps. Neuronal gain was calculated from the slope of the frequency–current (*F*–*I*) relationship using linear regression between 0 pA and the current step that produced the maximal firing frequency.

*Action potential properties* were quantified from the first spike elicited during depolarizing current injection. Latency to first spike was defined as the time from the onset of current injection to the peak of the first action potential. Phase-plane plots were generated by plotting the rate of change of voltage (dV/dt) against membrane voltage, from which the maximum and minimum dV/dt were obtained. Spike threshold was defined as the membrane potential at which dV/dt exceeded 30 mV/ms, and action potential peak corresponded to the maximum voltage reached during the spike.

Electrophysiological data were analyzed using a combination of Clampfit, Origin, and WinEDR. Current-clamp recordings were initially analyzed in Clampfit to extract basic membrane and firing properties. For more detailed analyses, including time constant estimation and phase-plane characterization, selected traces were exported as time–voltage data and further processed in Origin. Voltage-clamp recordings were analyzed in WinEDR, where spontaneous synaptic events were detected over a 2 min recording window using a template-matching function. Event frequency was calculated as the total number of detected events within this period, and additional kinetic parameters, including rise time, decay time, amplitude, and area, were extracted using the same software.

*Exclusion criteria*: Cells were initially selected for recording based on laminar position and pyramidal-like morphology. However, cells that displayed intrinsic electrophysiological properties characteristic of interneurons, including high-frequency firing, narrow action potential width, and more hyperpolarized RMP, were excluded from the analysis. For neurons included in the intrinsic electrophysiological analysis, successful biocytin recovery allowing post hoc confirmation of dendritic branching patterns was achieved in 73% of S1 neurons and 82% of V1 neurons.

### Statistics

All statistical analysis was performed using the GraphPad Prism 10.0.0 and Origin pro software.

All values are given as mean values ± standard error of the mean (SEM) if not indicated otherwise. The data obtained were first assessed for normal distribution using Shapiro–Wilk and Anderson Darling tests and then according to their distribution unpaired *t* test with Welch correction or Mann–Whitney *U* test were used to compare means. Further comparison of datasets was performed by using a nested *t* test to confirm whether the effects are spread out evenly in all samples/animals. A summary of all data comparisons with *p* value and statistical tests used is available in data tables and attached excel file.

## Results

### L2/3 PyNs exhibit distinct electrophysiological and morphological properties in S1 and V1

**Figure 1.**
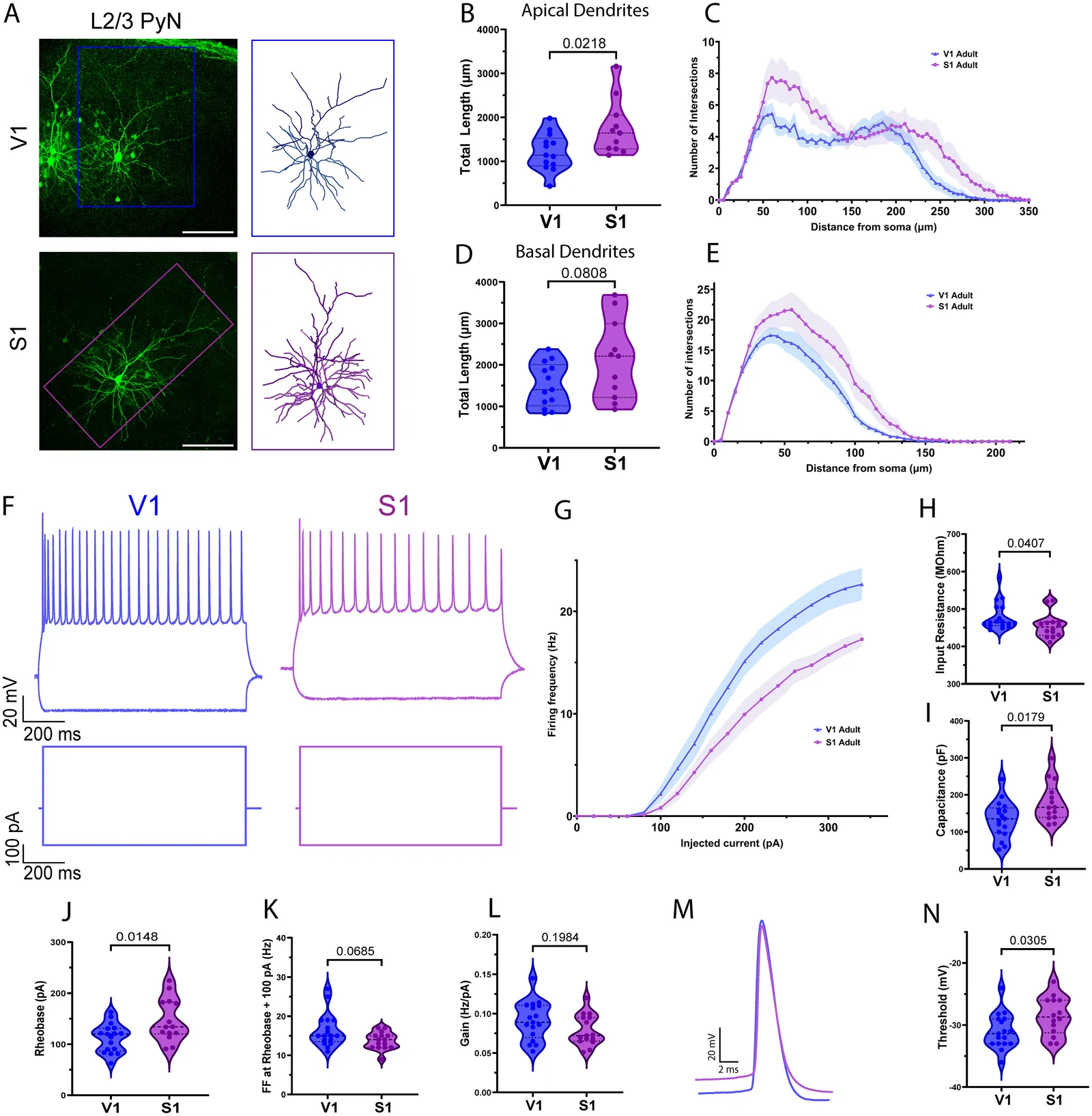
L2/3 PyNs exhibit distinct electrophysiological and morphological properties in S1 and V1. ***A***, Biocytin-filled adult V1 and S1 L2/3 PyNs and their morphological reconstructions (scale bar, 100 μM). ***B***, ***C***, Morphological quantification, total apical dendritic length and Sholl analysis of V1 versus S1 neurons [V1 = 1,215 ± 117 µm (*n* = 13 neurons; *N* = 7 mice); S1 = 1,752 ± 188 µm (*n* = 11 neurons; *N* = 6 mice); *p* = 0.0027/0.029, *t* test/nested *t* test]. ***D***, ***E***, Morphological quantification, total basal dendritic length and Sholl analysis of V1 versus S1 neurons [V1 = 1,517 ± 147 µm (*n* = 13 neurons; *N* = 7 mice); S1 = 2,124 ± 288 µm (*n* = 11 neurons; *N* = 6 mice); *p* = 0.08/0.1, *t* test/nested *t* test]. ***F***, Representative voltage traces of V1 and S1 neurons against −200 and 340 pA current injections. ***G***, Mean–SEM plot showing firing rates of neurons from V1 and S1 plotted against current injection in picoampere. ***H***, Violin plot showing IR of V1 versus S1 neurons [V1 = 479.81 ± 9.12 MΩ (*n* = 17 neurons; *N* = 7 mice); S1 = 455.128 ± 8.81 MΩ, (*n* = 14 neurons; *N* = 6 mice); *p* = 0.04/0.08, Mann–Whitney *U* test/nested *t* test]. ***I***, Violin plots showing capacitance of V1 versus S1 neurons [V1 = 133.6 ± 12.0 pF (*n* = 17 neurons; *N* = 7 mice); S1 = 180.47 ± 14.2 pF (*n* = 14 neurons; *N* = 6 mice); *p* = 0.018/0.02, *t* test/nested *t* test]. ***J–L***, Violin plots showing rheobase, firing frequency at Rb + 100 pA, and gain (from left to right) of V1 versus S1 neurons [V1 = 112.4 ± 6.75 pF (*n* = 17 neurons; *N* = 7 mice); S1 = 147.13 ± 11.2 pF (*n* = 14 neurons; *N* = 6 mice); *p* = 0.015/0.03, *t* test/nested *t* test], [V1 = 16.41 ± 1.05 Hz (*n* = 17 neurons; *N* = 7 mice); S1 = 13.73 ± 0.565 Hz (*n* = 15 neurons; *N* = 6 mice); *p* = 0.069/0.05, Mann–Whitney *U* test/nested *t* test], [V1 = 0.091 ± 0.00590 (*n* = 17 neurons, *N* = 7 mice); S1 = 0.079 ± 0.00508 Hz/pA (*n* = 15 neurons; *N* = 6 mice); *p* = 0.19/0.15, Mann–Whitney *U* test/nested *t* test]. ***M***, Representative action potential from V1 and S1 neurons. ***N***, Violin plot showing AP threshold of V1 versus S1 neurons [V1 = −31.00 ± 0.687 mV (*n* = 17 neurons; *N* = 7 mice); S1 = −28.5 ± 0.819 mV (*n* = 14 neurons; *N* = 6 mice); *p* = 0.03/0.11, *t* test/nested *t* test; blue V1; purple S1]. Refer to Extended Data Figure 1-1 for additional examples of biocytin-filled reconstructed neurons and Extended Data Figure 1-2 for additional electrophysiological properties and phase-plane plots (first AP), for example, S1 and V1 neurons. A summary of all statistical comparisons is provided in Table 1.

To determine whether L2/3 PyNs exhibit area-specific properties, we compared neurons in primary somatosensory barrel field (S1) and primary visual cortex (V1), two regions that process distinct sensory inputs. Whole-cell patch–clamp recordings were obtained from acute brain slices of adult (∼2-month-old) mice, and the neurons were filled with biocytin to enable post hoc morphological reconstruction. Since basal and apical dendrites integrate distinct sources of synaptic input (Lefort et al., 2009; Petreanu et al., 2009), we analyzed these compartments separately by measuring total dendritic length and branching complexity. Compared with S1 L2/3 PyNs, V1 L2/3 PyNs had significantly shorter apical dendrites, while basal dendrites showed a similar trend that did not reach significance. Sholl analysis further revealed reduced branching complexity in both apical and basal dendritic compartments of V1 L2/3 PyNs compared with S1 L2/3 PyNs (Fig. 1*A*–*E*). We confirmed that the relative depth of the neurons from the pial surface in the two areas was similar (Extended Data Fig. 1-1), indicating that the observed morphological differences were not due to sampling bias within L2/3 (Weiler et al., 2023).

10.1523/ENEURO.0103-26.2026.f1-1 Figure 1-1 **Layer 2/3 Pyramidal Neurons Exhibit Distinct Electrophysiological and Morphological Properties in V1 and S1**. Reconstructed dendritic morphologies of L2/3 pyramidal neurons with their Normalized cortical depth in the cortex [Normalized cortical depth = distance of neuronal soma from pial surface/ distance from Pial surface to end of Layer 6] of A:V1 and B: S1. Download Figure 1-1, TIF file.

**Figure 2.**
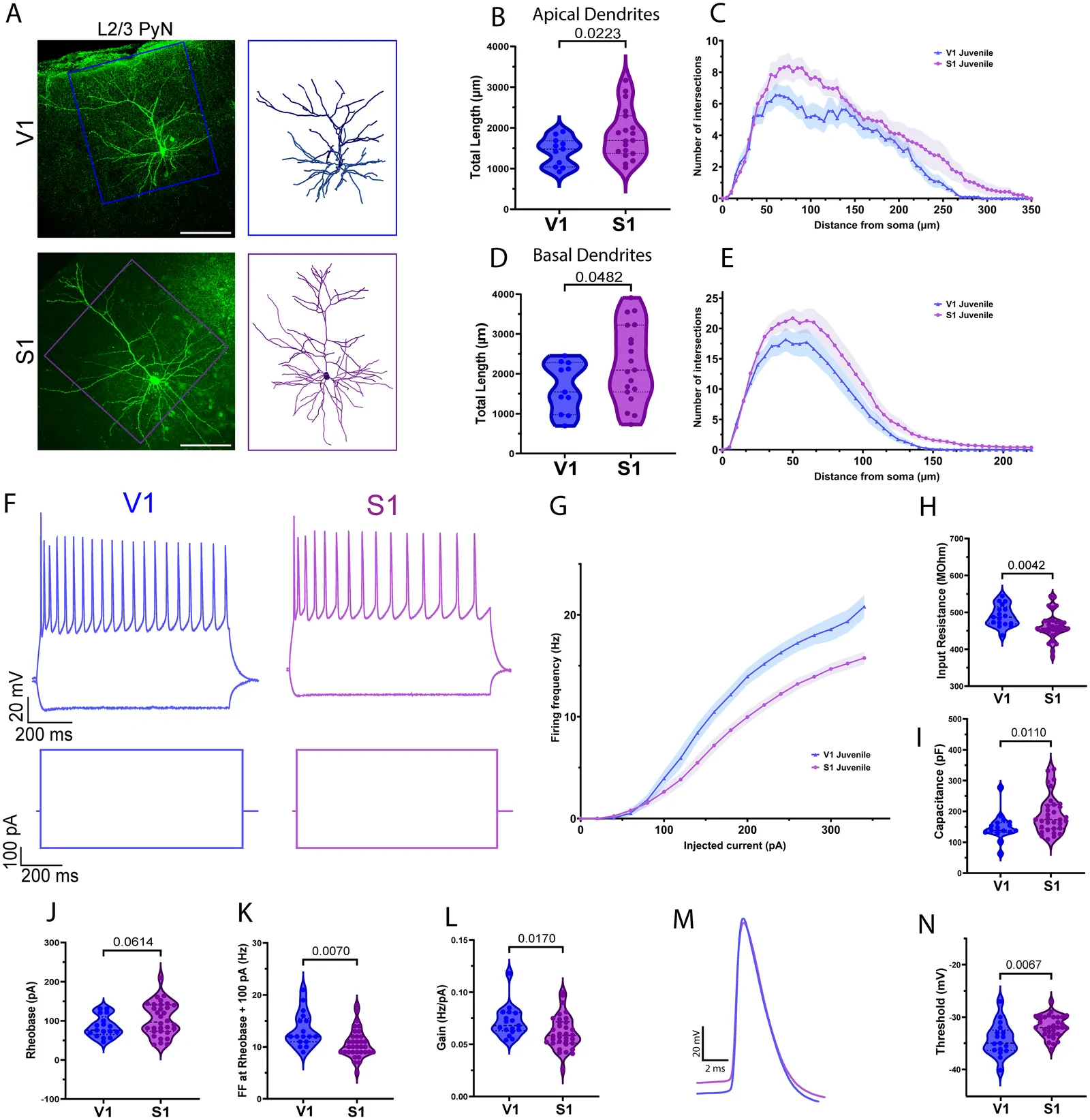
Early emergence of area-specific morphological and electrophysiological properties. ***A***, Biocytin-filled Juvenile V1 and S1 L2/3 PyNs and their morphological reconstructions (scale bar, 100 μM). ***B***, ***C***, Morphological quantification, total apical dendritic length and Sholl analysis of V1 versus S1 neurons [V1 = 1,421 ± 104 µm (*n* = 11 neurons; *N* = 6 mice); S1 = 1,846 ± 142 µm (*n* = 19 neurons; *N* = 6 mice); *p* = 0.022/0.06, *t* test/Nested *t* test]. ***D***, ***E***, Morphological quantification, total basal dendritic length and Sholl analysis of V1 versus S1 neurons [V1 = 1,658 ± 188 µm (*n* = 11 neurons; *N* = 5 mice); S1 = 2,257 ± 221 µm (*n* = 15 neurons; *N* = 6 mice); *p* = 0.048/0.06, *t* test/nested *t* test]. ***F***, Representative voltage traces of V1 and S1 neurons against −200 and 340 pA current injections. ***G***, Mean–SEM plot showing firing rates of neurons from V1 and S1 plotted against current injection in picoampere. ***H***, Violin plots showing IR of V1 versus S1 neurons [V1 = 490.75 ± 7.09 MΩ (*n* = 17 neurons; *N* = 6 mice); S1 = 461.50 ± 6.53 MΩ (*n* = 31 neurons; *N* = 11 mice); *p* = 0.004/0.035, *t* test/Nested *t* test]. ***I***, Violin plot showing capacitance of V1 versus S1 neurons [V1 = 148.61 ± 10.5 pF (*n* = 17 neurons; *N* = 6 mice); S1 = 190.52 ± 11.0 pF (*n* = 31 neurons; *N* = 11 mice); *p* = 0.011/0.1, Mann–Whitney *U* test/nested *t* test]. ***J–L***, Violin plots showing rheobase, firing frequency at Rb + 100 pA, and gain (from left to right) of V1 versus S1 neurons [V1 = 85.5 ± 6.56 pA (*n* = 17 neurons; *N* = 6 mice); S1 = 104.44 ± 7.48 pA (*n* = 31 neurons; *N* = 11 mice); *p* = 0.061/0.1, *t* test/Nested *t* test], [V1 = 13.352 ± 0.809 Hz (*n* = 17 neurons; *N* = 6 mice); S1 = 10.617 ± 0.474 Hz, (*n* = 33 neurons; *N* = 11 mice); *p* = 0.007/0.02, *t* test/Nested *t* test], [V1 = 0.0718 ± 0.00367 (*n* = 17 neurons; *N* = 6 mice); S1 = 0.0613 ± 0.00279 Hz/pA (*n* = 33 neurons; *N* = 11 mice); *p* = 0.017/0.047, Mann–Whitney *U* test/nested *t* test]. ***M***, Representative action potential from V1 and S1 neurons. ***N***, Violin plot showing AP threshold of S1 versus V1 neurons [V1 = −34.27 ± 0.764 (*n* = 17 neurons; *N* = 6 mice); S1 = −31.75 ± 0.363 Hz/pA, (*n* = 31 neurons; *N* = 11 mice); *p* = 0.006/0.1, Mann–Whitney *U* test/−31.75 ± 0.363; blue V1; purple S1]. Refer to Extended Data Figure 2-1 for additional electrophysiological properties and phase-plane plots (first AP), for example, juvenile S1 and V1 neurons. A summary of all statistical comparisons is provided in Table 2. Refer to Extended Data Figure 2-2 for comparison of spontaneous excitatory events between S1 and V1 L2/3 PyNs in juvenile mice.

10.1523/ENEURO.0103-26.2026.f1-2 Figure 1-2 **Layer 2/3 Pyramidal Neurons Exhibit Distinct Electrophysiological and Morphological Properties in V1 and S1**. Violin plots showing **A.** Resting membrane potential (V1 = -71.14 ± 1.38 mV [n = 17 neurons, N = 7 mice]; S1 = -68.14 ± 1.35 [n = 15 neurons, N = 6 mice]; p = 0.13, t-test; p = 0.16, Nested t-test); **B.** Sag ratio (V1 = 1.063 ± 0.203 [n = 17 neurons, N = 7 mice]; S1 = 0.988 ± 0.109 [n = 14 neurons, N = 6 mice]; p = 0.54, Mann-Whitney U test; p = 0.79, Nested t-test); **C.** Time constant (V1 = 18.06 ± 1.40 ms [n = 17 neurons, N = 7 mice]; S1 = 23.83 ± 2.28 [n = 14 neurons, N = 6 mice]; p = 0.032, Mann-Whitney U test; p = 0.047, Nested t-test); **D.** Maximum dV/dt (V1 = 158.28 ± 9.02 mV/ms [n = 17 neurons, N = 7 mice]; S1 = 147.33 ± 7.29 mV/ms [n = 14 neurons, N = 6 mice]; p = 0.35, t-test; p = 0.43, Nested t-test); **E**. Minimum dV/dt (V1 = -33.086 ± 1.15 mV/ms [n = 17 neurons, N = 7 mice]; S1 = -36.977 ± 1.19 mV/ms [n = 14 neurons, N = 6 mice]; p = 0.026, t-test; p = 0.037, Nested t-test); **F.** Peak Action Potential amplitude (V1 = 128.7035 ± 2.72 pA [n = 17 neurons, N = 7 mice]; S1 = 128.701 ± 1.20 pA [n = 14 neurons, N = 6 mice]; p = 0.20, Mann-Whitney U test; p = 0.99, Nested t-test); **G**. Latency to first spike (V1 = 0.2843 ± 0.0542 s [n = 17 neurons, N = 7 mice]; S1 = 0.19071 ± 0.0339 s [n = 14 neurons, N = 6 mice]; p = 0.14, Mann-Whitney U test; p = 0.99, Nested t-test), of adult L2/3 PyNs V1 and S1 neurons. **H**. Neurons plotted in 3D space showing spatial distribution based on intrinsic electrophysiological properties as on axis (Normalized values; Z score). **I.** Three faces, each show two of the three axis of the plot in H. **J-K**. Phase plane plots for first action potential from example neurons recorded from adult mice, J: V1 (Blue) and K: S1 (Purple). Download Figure 1-2, TIF file.

Although both populations displayed comparable RMP (Extended Data Fig. 1-2*A*), their passive membrane properties reflected their morphological differences. Consistent with their reduced physical size, V1 PyNs displayed significantly higher IR and lower membrane capacitance than S1 neurons (Fig. 1*F*,*H*,*I*; also see Table 1), while no changes were observed in their sag ratio (Extended Data Fig. 1-2*B*). Additionally, differences in the membrane time constant (Extended Data Fig. 1-2*C*) suggest that V1 neurons are optimized for high-gain responses to synaptic input, which is consistent with reduced membrane surface area. As a result, V1 neurons fired more action potentials than S1 neurons in response to equivalent current injections (Fig. 1*F*,*G*,*K*). Rheobase and action potential thresholds were significantly lower in V1 neurons (Fig. 1*J*,*M*,*N*), and the gain showed a similar trend (Fig. 1*L*), while the minimum dV/dt was larger (Extended Data Fig. 1-2*E*), indicating greater intrinsic excitability of V1 neurons relative to S1 PyNs. Properties such as peak amplitude of action potential and latency to first spike did not show any significant differences (Extended Data Fig. 1-2*F*,*G*). Additionally, we plotted individual neurons in a three-dimensional space defined by selected electrophysiological parameters. The neurons from the two cortical areas showed a moderate separation when plotted in 3D using three significantly different parameters between S1 and V1 consisting of both sub- and suprathreshold properties (Extended Data Fig. 1-2*H*,*I*).

**Table 1.** Morphological and electrophysiological properties of Adult V1 versus S1 L2/3 PyNs

	Property	V1	S1	*p* value/test	*p* value (nested)
A. Dendritic morphology					
1	Total apical dendrite length (µm)	1,215 ± 117 (*n* = 13 neurons; *N* = 7 mice)	1,752 ± 188 (*n* = 11 neurons; *N* = 6 mice)	0.027/*T* test	0.029
2	Total basal dendrite length (µm)	1,517 ± 147 (*n* = 13 neurons; *N* = 7 mice)	2,124 ± 288 (*n* = 11 neurons; *N* = 6 mice)	0.08/*T* test	0.1
B. Subthreshold properties					
1	RMP (mV)	−71.14 ± 1.38 (*n* = 17 neurons; *N* = 7 mice)	−68.14 ± 1.35 (*n* = 15 neurons; *N* = 6 mice)	0.131/*T* test	0.16
2	IR (MΩ)	479.81 ± 9.12 (*n* = 17 neurons; *N* = 7 mice)	455.128 ± 8.81 (*n* = 14 neurons; *N* = 6 mice)	0.041/Mann–Whitney *U* test	0.08
3	Membrane time constant (ms)	18.06 ± 1.40 (*n* = 17 neurons; *N* = 7 mice)	23.83 ± 2.28 (*n* = 14 neurons; *N* = 6 mice)	0.033/Mann–Whitney *U* test	0.047
4	Input capacitance (pF)	133.6 ± 12.0 (*n* = 17 neurons; *N* = 7 mice)	180.47 ± 14.2 (*n* = 14 neurons; *N* = 6 mice)	0.018/*T* test	0.02
5	Sag ratio	1.063 ± 0.203 (*n* = 17 neurons; *N* = 7 mice)	0.988 ± 0.109 (*n* = 14 neurons; *N* = 6 mice)	0.54/Mann–Whitney *U* test	0.79
C. Suprathreshold properties					
1	Rheobase (pA)	112.41 ± 6.75 (*n* = 17 neurons; *N* = 7 mice)	147.13 ± 11.2 (*n* = 14 neurons; *N* = 6 mice)	0.0148/*T* test	0.03
2	Threshold (mV)	−31.00 ± 0.687 (*n* = 17 neurons; *N* = 7 mice)	−28.5 ± 0.819 (*n* = 14 neurons; *N* = 6 mice)	0.03/*T* test	0.11
3	Gain (Hz/pA)	0.091 ± 0.00590 (*n* = 17 neurons; *N* = 7 mice)	0.079 ± 0.00508 (*n* = 15 neurons; *N* = 6 mice)	0.19/Mann–Whitney *U* test	0.15
4	FF at rheobase + 100 pA (Hz)	16.41 ± 1.05 (*n* = 17 neurons; *N* = 7 mice)	13.73 ± 0.565 (*n* = 15 neurons; *N* = 6 mice)	0.069/Mann–Whitney *U* test	0.05
5	Maximum dV/dt (mV/ms)	158.28 ± 9.02 (*n* = 17 neurons; *N* = 7 mice)	147.33 ± 7.29 (*n* = 14 neurons; *N* = 6 mice)	0.35/*T* test	0.43
6	Minimum dV/dt (mV/ms)	−33.086 ± 1.15 (*n* = 17 neurons; *N* = 7 mice)	−36.977 ± 1.19 (*n* = 14 neurons; *N* = 6 mice)	0.026/*T* test	0.037
7	Maximum AP peak (mV)	128.7035 ± 2.72 (*n* = 17 neurons; *N* = 7 mice)	128.701 ± 1.20 (*n* = 14 neurons; *N* = 6 mice)	0.2/Mann–Whitney *U* test	0.99
8	Latency to first spike (s)	0.2843 ± 0.0542 (*n* = 17 neurons; *N* = 7 mice)	0.19071 ± 0.0339 (*n* = 14 neurons; *N* = 6 mice)	0.14/Mann–Whitney *U* test	0.99

Values are given as mean ± SEM.

Together, these findings demonstrate that L2/3 PyNs differ systematically across these two sensory cortical areas: neurons in V1 exhibit reduced dendritic complexity accompanied by higher IR and increased intrinsic excitability, whereas neurons in S1 show larger dendritic arbors and comparatively lower excitability.

### Early emergence of area-specific morphological and electrophysiological properties

To determine whether the observed differences manifest before adulthood, we examined L2/3 PyNs in juvenile (∼1 month old) mice, a stage where S1 maturation is complete, whereas V1 is still maturing (Espinosa and Stryker, 2012). Morphological analysis in juvenile mice revealed a similar pattern as that in adult mice such that S1 PyNs exhibited larger and more highly branched dendritic arbors, whereas V1 neurons remained smaller and less complex, both in apical and basal dendritic structure (Fig. 2*A*–*E*). Thus, the areal differences in dendritic structure are already evident at the juvenile stage. We next assessed their intrinsic electrophysiological properties. V1 neurons exhibited a more hyperpolarized membrane potential (Extended Data Fig. 2-1*A*), higher IR (Fig. 2*H*), lower membrane capacitance (Fig. 2*I*), and a trend toward shorter membrane time constant (Extended Data Fig. 2-1*B*) relative to S1 neurons. V1 neurons also displayed greater excitability and number of action potentials (Fig. 2*F*,*G*,*J*–*N*; also see Table 2). Similar to adult mice, we plotted individual neurons in a three-dimensional space defined by selected electrophysiological parameters same as in adult mice, and the two cortical areas showed a moderate clustering in this space revealing their separability (Extended Data Fig. 2-1*H*,*I*).

10.1523/ENEURO.0103-26.2026.f2-1 Figure 2-1 **Early Emergence of Area-Specific Morphological and Electrophysiological Properties.** Violin plots showing **A.** Resting membrane potential (V1 = -73.6 ± 1.07 mV [n = 17 neurons, N = 6 mice]; S1 = -68.32 ± 0.777 [n = 34 neurons, N = 11 mice]; p = 0.0003, Mann-Whitney U test; p = 0.04, Nested t-test); **B.** Time constant (V1 = 21.94 ± 2.41 ms [n = 17 neurons, N = 6 mice]; S1 = 26.24 ± 1.83 ms [n = 31 neurons, N = 11 mice]; p = 0.08, Mann-Whitney U test; p = 0.16, Nested t-test); **C.** Latency to first spike (V1 = 0.29 ± 0.039 s [n = 17 neurons, N = 6 mice]; S1 = 0.199 ± 0.013 s [n = 31 neurons, N = 11 mice]; p = 0.62, Mann-Whitney U test;p = 0.2, Nested t-test); **D.** Maximum dV/dt (V1 = 160.264 ± 7.25 mV/ms [n = 17 neurons, N = 6 mice]; S1 = 144.854 ± 4.55 mV/ms [n = 31 neurons, N = 11 mice]; p = 0.08, t-test; p = 0.16, Nested t-test); **E.** Minimum dV/dt (V1 = -27.38 ± 0.93 mV/ms [n = 17 neurons, N = 6 mice]; S1 = -27.37 ± 0.98 mV/ms [n = 31 neurons, N = 11 mice]; p = 0.99, t-test; p = 0.79, Nested t-test); **F.** Peak Action Potential amplitude (V1 = 126.40 ± 2.15 pA [n = 17 neurons, N = 6 mice]; S1 = 125.99 ± 1.40 pA [n = 33 neurons, N = 11 mice]; p = 0.97, Mann-Whitney U test; p = 0.7, Nested t-test); **G.** Sag ratio (V1 = 1.57 ± 0.18 [n = 17 neurons, N = 6 mice]; S1 = 1.33 ± 0.1 [n = 31 neurons, N = 11 mice]; p = 0.26, t-test; p = 0.33, Nested t-test), of adult L2/3 PyNs V1 and S1 neurons. **H.** Neurons plotted in 3D space showing spatial distribution based on intrinsic electrophysiological properties as on axis (Normalized values; Z score) **I.** Three faces of plot H, each show two of the three axis of the plot in H. **J-K.** Phase plane plots for first Action potential from each neuron recorded from juvenile mice. J: V1 (Blue) and K: S1 (Purple). Download Figure 2-1, TIF file.

10.1523/ENEURO.0103-26.2026.f2-2 Figure 2-2 **Comparison of spontaneous excitatory events between S1 and V1 L2/3 PyNs in juvenile mice. A.** Representative voltage clamp traces from S1(Purple) and V1(Blue) neurons. **B.** Violin plots showing event frequency on S1BF vs V1 neurons (V1 = 3.441 ± 0.38 events/s, [n = 15 neurons, N = 6 mice]; S1 = 2.7 ± 0.22 events/s [n = 27 neurons, N = 12 mice]; p = 0.069, Mann Whitney test). Cumulative frequency plots comparing: **C.** Event Rise time; **D.** Event Decay time; **E.** Event amplitude; **F.** Event area of S1BF and V1 neurons. Decay vs Rise time scatter plots for events for: **G.** S1BF and **H.** V1 neurons. Relative Frequency and violin plots comparing: **I.** Event Rise time (V1 = 2.68 ± 0.13 ms, [n = 15 neurons, N = 6 mice ]; S1 = 3.01 ± 0.16 ms [n = 27 neurons, N = 12 mice]; p = 0.11, Mann Whitney test); **J.** Event Decay time (V1 = 8.98 ± 0.53 ms, [n = 15 neurons, N = 6 mice ]; S1 = 9.9 ± 0.37 ms [n = 27 neurons, N = 12 mice]; p = 0.07, Mann Whitney test); **K.** Event amplitude (V1 = 7.5 ± 0.73 pA, [n = 15 neurons, N = 6 mice ]; S1 = 8.04 ± 0.36 pA [n = 27 neurons, N = 12 mice]; p = 0.15, Mann Whitney test); **L.** Event area (V1 = 60.56 ± 5.12 pA.ms, [n = 15 neurons, N = 6 mice ]; S1 = 79.89 ± 4.08 pA.ms [n = 27 neurons, N = 12 mice]; p = 0.002, Mann Whitney test) of S1 and V1 neurons. (All data points in violin plots are average per cell), (Purple - S1; Blue - V1). Download Figure 2-2, TIF file.

**Table 2.** Morphological and electrophysiological properties of Juvenile V1 versus S1 L2/3 PyNs

	Property	V1	S1	*p* value/test	*p* value (nested)
A. Dendritic morphology					
1	Total apical dendrite length (µm)	1,421 ± 104 (*n* = 11 neurons; *N* = 6 mice)	1,846 ± 142 (*n* = 19 neurons; *N* = 6 mice)	0.022/*T* test	0.06
2	Total basal dendrite length (µm)	1,658 ± 188 (*n* = 11 neurons; *N* = 5 mice)	2,257 ± 221 (*n* = 15 neurons; *N* = 5 mice)	0.048/*T* test	0.06
B. Subthreshold properties					
1	RMP (mV)	−73.6 ± 1.07 (*n* = 17 neurons; *N* = 6 mice)	−68.32 ± 0.777 (*n* = 34 neurons; *N* = 11 mice)	0.0003/Mann–Whitney *U* test	0.04
2	IR (MΩ)	490.75 ± 7.09 (*n* = 17 neurons; *N* = 6 mice)	461.50 ± 6.53 (*n* = 31 neurons; *N* = 11 mice)	0.004/*T* test	0.035
3	Membrane time constant (ms)	21.94 ± 2.41 (*n* = 17 neurons; *N* = 6 mice)	26.24 ± 1.83 (*n* = 31 neurons; *N* = 11 mice)	0.082/Mann–Whitney *U* test	0.16
4	Input capacitance (pF)	148.61 ± 10.5 (*n* = 17 neurons; *N* = 6 mice)	190.52 ± 11.0 (*n* = 31 neurons; *N* = 11 mice)	0.011/Mann–Whitney *U* test	0.13
5	Sag ratio	1.57484 ± 0.18 (*n* = 17 neurons; *N* = 6 mice)	1.3349 ± 0.1 (*n* = 31 neurons; *N* = 11 mice)	0.26/*T* test	0.33
C. Suprathreshold properties					
1	Rheobase (pA)	85.5 ± 6.56 (*n* = 17 neurons; *N* = 6 mice)	104.44 ± 7.48 (*n* = 31 neurons; *N* = 11 mice)	0.061/*T* test	0.11
2	Threshold (mV)	−34.27 ± 0.764 (*n* = 17 neurons; *N* = 6 mice)	−31.75 ± 0.363 (*n* = 31 neurons; *N* = 11 mice)	0.006/*T* test	0.11
3	Gain (Hz/pA)	0.0718 ± 0.00367 (*n* = 17 neurons; *N* = 6 mice)	0.0613 ± 0.00279 (*n* = 33 neurons; *N* = 11 mice)	0.017/Mann–Whitney *U* test	0.047
4	FF at rheobase + 100 pA (Hz)	13.352 ± 0.809 (*n* = 17 neurons; *N* = 6 mice)	10.617 ± 0.474 (*n* = 33 neurons; *N* = 11 mice)	0.007/*T* test	0.021
5	Maximum dV/dt (mV/ms)	160.264 ± 7.25 (*n* = 17 neurons; *N* = 6 mice)	144.854 ± 4.55 (*n* = 31 neurons; *N* = 11 mice)	0.082/*T* test	0.16
6	Minimum dV/dt (mV/ms)	−27.383 ± 0.929 (*n* = 17 neurons; *N* = 6 mice)	−27.37 ± 0.985 (*n* = 31 neurons; *N* = 11 mice)	0.99/*T* test	0.79
7	Maximum AP peak (mV)	126.40 ± 2.15 (*n* = 17 neurons; *N* = 6 mice)	125.99 ± 1.40 (*n* = 33 neurons; *N* = 11 mice)	0.97/Mann–Whitney *U* test	0.7
8	Latency to first spike (s)	0.29 ± 0.039 (*n* = 17 neurons; *N* = 6 mice)	0.199 ± 0.013 (*n* = 31 neurons; *N* = 11 mice)	0.62/Mann–Whitney *U* test	0.2

Values are given as mean ± SEM.

We sought to examine whether the observed morphological differences had an impact on the synaptic inputs received by V1 and S1 neurons. To that end, we recorded spontaneous excitatory postsynaptic currents using voltage clamp (Extended Data Fig. 2-2*A*). We observed that V1 neurons displayed a trend toward a higher frequency of events (Extended Data Fig. 2-2*B*), and these events were characterized by faster rise and decay kinetics and a significantly smaller charge transfer compared with S1 neurons (Extended Data Fig. 2-2). These findings indicate that synaptic input dynamics, like intrinsic membrane properties, differ between these sensory cortical areas at the juvenile stage. Although V1 neurons have less dendritic complexity, they showed a trend toward higher event frequency, which could reflect a higher density of synapses per unit dendritic length rather than a simple scaling with total dendritic length.

Together, these results indicate that the morphological and electrophysiological distinctions between S1 and V1 PyNs are already established by juvenile stages and persist into adulthood, suggesting that these areal specializations emerge early.

### Conditional deletion of *Lhx2* affects dendritic morphology in S1 but not in V1 PyNs

**Figure 3.**
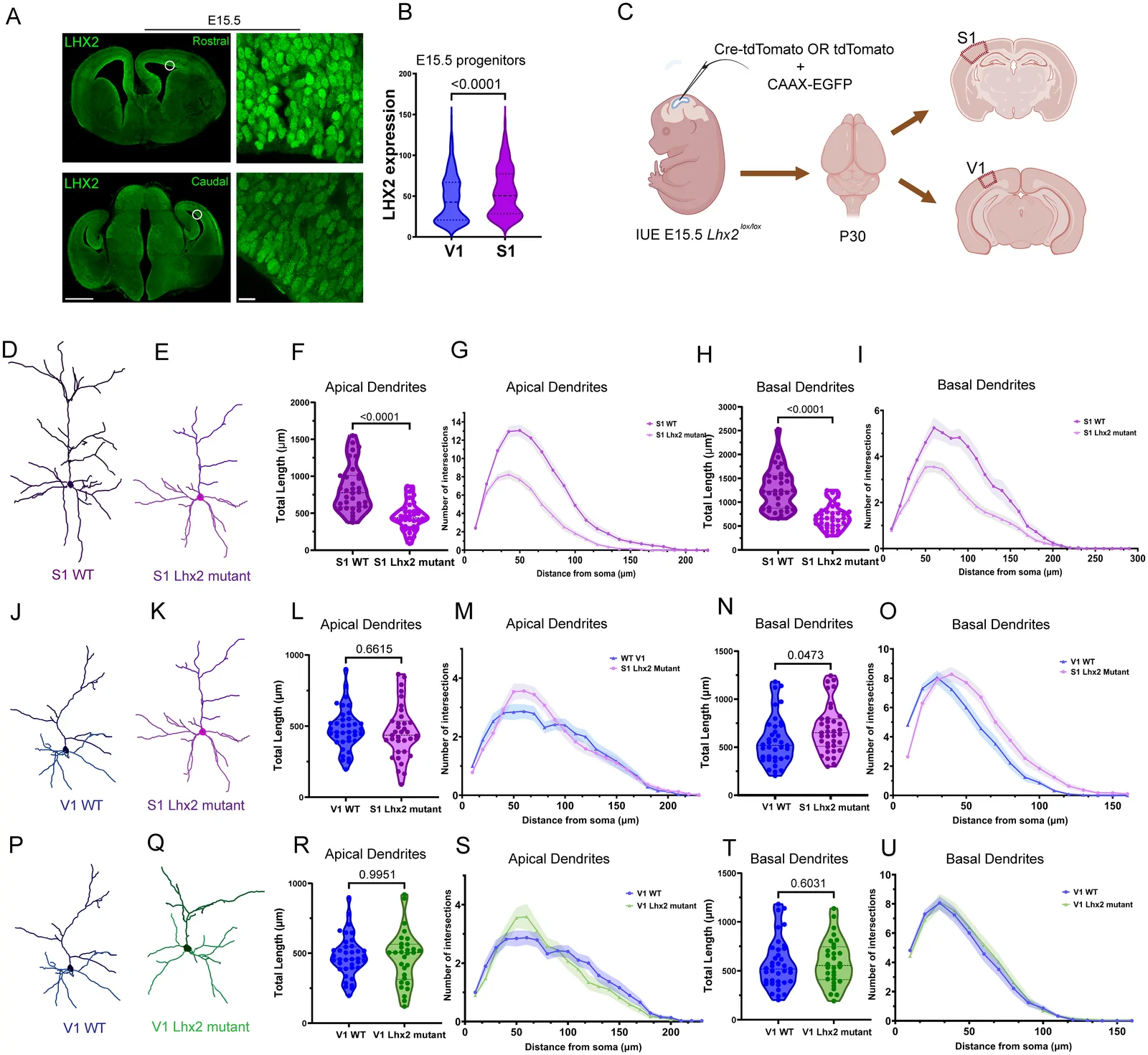
Conditional deletion of *Lhx2* affects dendritic morphology in S1 but not in V1 PyNs. ***A***, LHX2 immunostaining in E15.5 rostral and caudal sections. ROIs from S1 (rostral) and V1 (caudal) ventricular zone regions (marked by white circles) were imaged at high magnification as shown in the adjacent panels (scale bar, 750 µm (low mag) and 10 µm (high mag). ***B***, Violin plots comparing LHX2 levels in V1 versus S1 E15.5 progenitors [LHX2 expression (mean gray value) V1 = 46.56 ± 1.46 (*n* = 375 cells; *N* = 3 E15.5 embryos); S1 = 54.94 ± 1.50 (*n* = 385 cells; *N* = 3 E15.5 embryos); *p* = 0.0161, Nested *t* test]. ***C***, Schematic of conditional *Lhx2* deletion from E15.5 progenitors. Conditional mutant *Lhx2^lox/^*^lox^ mice were used, in which Cre-tdTomato or tdTomato were electroporated to generate *Lhx2* mutant and control neurons, respectively. Neurons were analyzed for morphology at P30. ***D***, ***E***, Representative dendritic morphology reconstruction of S1 WT and S1 Lhx2 mutant neuron. ***F–I***, Morphological quantification - Total apical and basal dendritic length and Sholl analysis of S1 WT versus S1 Lhx2 mutant neurons (reanalyzed from Bose et al., 2025; apical dendrite length [S1 WT = 831 ± 54.2 µm (*n* = 34 neurons; *N* = 4 mice); S1 Lhx2 mutant = 460 ± 31.3 µm (*n* = 35 neurons; *N* = 4 mice); *p* < 0.0001/0.01, Mann–Whitney *U* test/Nested *t* test]); basal dendrite length [S1 WT = 1,285 ± 75.2 µm (*n* = 34 neurons; *N* = 4 mice); S1 Lhx2 mutant = 688 ± 41 µm (*n* = 37 neurons; *N* = 4 mice); *p* > 0.001/0.007, Mann–Whitney *U* test/nested *t* test]. ***J***, ***K***, Representative dendritic morphology reconstruction of V1 WT and S1 Lhx2 mutant neuron. ***L–O***, Morphological quantification, total apical and basal dendritic length and Sholl analysis of V1 WT versus S1 Lhx2 mutant neurons (reanalyzed from Bose et al., 2025; apical dendrite length [V1 WT = 478 ± 23.8 µm (*n* = 38 neurons; *N* = 3 mice); S1 Lhx2 mutant = 460 ± 31.3 µm (*n* = 35 neurons; *N* = 4 mice); *p* = 0.66/0.88, *t* test/Nested *t* test]); basal dendrite length [V1 WT = 570 ± 41.6 µm (*n* = 37 neurons; *N* = 3 mice); S1 Lhx2 mutant = 688 ± 41 µm (*n* = 37 neurons; *N* = 4 mice); *p* = 0.047/0.3, *t* test/Nested *t* test]. ***P***, ***Q***, Representative dendritic morphology reconstruction of V1 WT and V1 Lhx2 mutant neuron. ***R–U***, Morphological quantification, total apical and basal dendritic length and Sholl analysis of V1 WT versus V1 Lhx2 mutant neurons [V1 WT = 478 ± 23.8 µm (*n* = 38 neurons; *N* = 3 mice); V1 Lhx2 mutant = 470 ± 34.9 µm (*n* = 30 neurons; *N* = 3 mice); *p* = 0.85/0.92, *t* test/Nested *t* test]; basal dendrite length [V1 WT = 570 ± 41.6 µm (*n* = 37 neurons; *N* = 3 mice); V1 Lhx2 mutant = 587 ± 43 µm (*n* = 30 neurons; *N* = 3 mice); *p* = 0.77/0.78, *t* test/Nested *t* test; purple S1 WT; blue V1 WT (wild type/control); light purple S1 Lhx2 mutant; light green V1 Lhx2 mutant]. Refer to Extended Data Figure 3-1 for low- and high-magnification images of the E15.5 caudal and rostral regions used for LHX2 expression comparisons and quantification of individual replicates and Extended Data Figure 3-2 for additional morphometric analysis on S1 LhX2 mutants compared with S1 and V1 WT neurons. Refer to Extended Data Figure 3-3 for morphometric comparisons between biocytin-filled neurons versus electroporated neurons. A summary of all statistical comparisons is provided in Table 3.

Since LHX2 is an established regulator of dendritic morphogenesis (Wang et al., 2017; Bose et al., 2025), we tested whether this transcription factor contributes to the structural differences observed between S1 and V1 PyNs. We first examined LHX2 levels at E15.5 and found S1 progenitors to display higher levels than V1 progenitors (Fig. 3*A*,*B*; Extended Data Fig. 3-1). In prior work, we demonstrated that conditional deletion of *Lhx2* in E15.5 S1 progenitors causes a striking shrinkage in dendritic length and arborization in the resulting L2/3 PyNs at the juvenile stage (Bose et al., 2025; Fig. 3*D*–*I*; Extended Data Fig. 3-2*A*,*B*).

10.1523/ENEURO.0103-26.2026.f3-1 Figure 3-1 **LHX2 expression across cortical regions at E15.5. A.** LHX2 immunostaining in E15.5 rostral (top) and caudal (bottom) sections. Region of Interests (ROI) from S1 (rostral) and V1 (caudal) ventricular zone regions (marked by white boxes) were imaged at higher magnification for quantification (scale bar = 750 µm (A) and 100 µm (B); **B.** Magnified regions from corresponding sections in A. **C.** Quantification of LHX2 expression in individual replicates. Nested comparisons of LHX2 levels in V1 vs S1 E15.5 progenitors (LHX2 expression (Mean Gray Value) V1 = 46.56 ± 1.46 [n = 375 cells, N = 3 E15.5 embryos]; S1 = 54.94 ± 1.50 [n = 385 cells, N = 3 E15.5 embryos]; p = 0.0161, Nested t-test). Download Figure 3-1, TIF file.

10.1523/ENEURO.0103-26.2026.f3-2 Figure 3-2 **Conditional Deletion of Lhx2 Affects Dendritic Morphology in S1 but not in V1 PyNs and Lhx2 overexpression is sufficient to enhance the dendritic arborization of V1 PyNs. A-B**. Nested Total dendritic length of S1 and S1 Lhx2 mutant L2/3 PyNs (From Bose et al 2025) of Apical (S1 WT = 831 ± 54.7 µm [n = 34 neurons, N = 4 mice]; S1 Lhx2 mutant = 460 ± 31.2 µm [n = 35 neurons, N = 4 mice]; p = 0.01, Nested t-test) and basal dendrites (S1 WT = 1285 ± 75.2 µm [n = 34 neurons, N = 4 mice]; S1 Lhx2 mutant = 687 ± 41 µm [n = 37 neurons, N = 4 mice]; p = 0.007, Nested t-test). Morphological analysis - Nested Total dendritic length and Sholl analysis: **C-D.** Apical dendrites (V1 Juvenile = 478 ± 23.8 µm [n = 38 neurons, N = 3 mice]; S1 Juvenile = 831 ± 54.3 µm [n = 34 neurons, N = 4 mice]; p = 0.01, Nested t-test) and **E-F.** Basal dendrites (V1 Juvenile = 570 ± 41.6 µm [n = 37 neurons, N = 3 mice]; S1 Juvenile = 1285 ± 75.2 µm [n = 34 neurons, N = 4 mice]; p = 0.004, Nested t-test) of V1 and S1 L2/3 PyNs labelled by CAAX-EGFP via in-utero electroporation of E15.5 embryo of Lhx2lox/lox mice. **G-H.** Total dendritic length of V1 WT and S1 Lhx2 mutant L2/3 PyNs of Apical (WT V1 = 478 ± 23.8 µm [n = 38 neurons, N = 3 mice]; S1 Lhx2 mutant = 460 ± 31.3 µm [n = 35 neurons, N = 4 mice]; p = 0.88, Nested t-test) and basal dendrites (WT V1 = 570 ± 41.6 µm [n = 37 neurons, N = 3 mice]; S1 Lhx2 mutant = 688 ± 41 µm [n = 37 neurons, N = 4 mice]; p = 0.3, Nested t-test). **I-J.** Nested Total dendritic length of V1 WT and V1 Lhx2 mutant L2/3 PyNs of Apical (WT V1 = 478 ± 23.8 µm [n = 38 neurons, N = 3 mice]; V1 Lhx2 mutant = 470 ± 34.9 µm [n = 30 neurons, N = 3 mice]; p = 0.92, Nested t-test) and basal dendrites (WT V1 = 570 ± 41.6 µm [n = 37 neurons, N = 3 mice]; V1 Lhx2 mutant = 587 ± 43 µm [n = 30 neurons, N = 3 mice]; p = 0.78, Nested t-test). L, M: Total dendritic length of V1 WT and V1 Lhx2 OE L2/3 PyNs of apical (V1 WT = 651 ± 45.5 µm, [n = 23 neurons, N = 4 mice]; V1 Lhx2 OE = 651 ± 64.5 µm [n = 18 neurons, N = 4 mice]; p = 0.99, Nested t-test) and basal dendrites (V1 WT = 853 ± 36.2 µm, [n = 23 neurons, N = 4 mice]; V1 Lhx2 OE = 1014 ± 45.6 µm [n = 21 neurons, N = 4 mice]; p = 0.031, Nested t-test). Download Figure 3-2, TIF file.

10.1523/ENEURO.0103-26.2026.f3-3 Figure 3-3 **Morphological comparison of S1 neurons obtained by biocytin filled neurons [S1 (Biocytin)] and by in-utero electroporation of CAAX-EGFP in E15.5 progenitors [S1 (EP)]. A.** Comparison of total Apical dendrite length (S1 (Biocytin) = 1846 ± 142 µm [n = 19 neurons, N = 6 mice]; S1 (EP) = 831 ± 54.7 µm [n = 34 neurons, N = 4 mice]; 587 ± 43 µm [n = 30 neurons, N = 3 mice]; p = 0.0024, Nested t-test). **B.** Total Basal dendrite length (S1 (Biocytin) = 2257 ± 221 µm [n = 15 neurons, N = 6 mice]; S1 (EP) = 1285 ± 75.2 µm [n = 34 neurons, N = 4 mice]; p = 0.015, Nested t-test). Download Figure 3-3, TIF file.

We used IUE of a construct encoding a membrane-tethered EGFP (CAAX-EGFP) in E15.5 progenitors to label L2/3 neurons (Fig. 3*C*). When examined in juvenile mice, we found that electroporated S1 and V1 PyNs displayed dendritic morphologies that recapitulated the differences (Extended Data Fig. 3-2*C–F*) we found in biocytin-filled neurons (Fig. 1).

Interestingly, we found that control V1 PyNs were morphologically similar to S1 *Lhx2* mutant PyNs we had reported in our previous study. Apical and basal dendrite length and branching was not significantly different from control V1 PyNs compared with *Lhx2* mutant S1 PyNs (Fig. 3*J*–*O*; Extended Data Fig. 3-2*G*,*H*; Bose et al., 2025; also see Table 3).

**Table 3.** Dendritic morphology of S1 wild type versus S1 Lhx2 mutant and S1 (biocytin) versus S1 (EP) L2/3 PyNs (data from Bose et al, 2025)

	Property	S1 WT	S1 Lhx2 mutant	*p* value/test	*p* value (nested)
A. Dendritic morphology					
1	Total apical dendrite length (µm)	831 ± 54.7 (*n* = 34 neurons; *N* = 4 mice)	460 ± 31.3 (*n* = 35 neurons; *N* = 4 mice)	0.0001/Mann–Whitney *U* test	0.01
2	Total basal dendrite length (µm)	1,285 ± 75.2 (*n* = 34 neurons; *N* = 4 mice)	688 ± 41 (*n* = 37 neurons; *N* = 4 mice)	0.0001/Mann–Whitney *U* test	0.007
		S1 (biocytin)	S1 (EP)		
3	Total apical dendrite length (µm)	1,846 ± 142 (*n* = 19 neurons; *N* = 6 mice)	831 ± 54.7 (*n* = 34 neurons; *N* = 4 mice)	0.0001/Mann–Whitney *U* test	0.0024
4	Total basal dendrite length (µm)	2,257 ± 221 (*n* = 15 neurons; *N* = 5 mice)	1,285 ± 75.2 (*n* = 34 neurons; *N* = 4 mice)	0.0001/Mann–Whitney *U* test	0.0015

Values are given as mean ± SEM.

Based on this, we hypothesized that loss of *Lhx2* would result in a shrinkage of V1 PyNs rendering them smaller than the corresponding V1 controls. We coelectroporated a plasmid encoding tdTomato (control) or Cre-tdTomato, together with CAAX-EGFP at E15.5 in *Lhx2^lox/lox^* embryos and examined the dendritic morphology of the resulting L2/3 PyNs in juvenile animals (Fig. 3*C*). In contrast to our previous findings in S1, deletion of *Lhx2* in V1 progenitors did not alter the dendritic architecture of V1 PyNs. Quantitative analysis revealed no significant differences in total dendritic length or arborization between apical or basal dendrites of control and *Lhx2* mutant neurons (Fig. 3*P*–*U*; Extended Data Fig. 3-2*I–J*; also see Table 4).

**Table 4.** Dendritic morphologies of V1 WT versus S1 Lhx2 mutants; V1 Lhx2 mutants; V1 Lhx2 overexpression

	Property	V1 WT	S1 WT	*p* value/test	*p* value (nested)
A. Dendritic morphology					
1	Total apical dendrite length (µm)	478 ± 23.8 (*n* = 38 neurons; *N* = 3 mice)	831 ± 54.7 (*n* = 34 neurons; *N* = 4 mice)	0.0001/*T* test	0.01
2	Total basal dendrite length (µm)	570 ± 41.6 (*n* = 37 neurons; *N* = 3 mice)	1,285 ± 75.2 (*n* = 34 neurons; *N* = 4 mice)	0.0001/*T* test	0.004
		V1 WT	S1 Lhx2 mutant	*p* value/test	*p* value (nested)
3	Total apical dendrite length (µm)	478 ± 23.8 (*n* = 38 neurons; *N* = 3 mice)	460 ± 31.3 (*n* = 35 neurons; *N* = 4 mice)	0.66/*T* test	0.88
4	Total basal dendrite length (µm)	570 ± 41.6 (*n* = 37 neurons; *N* = 3 mice)	688 ± 41 (*n* = 37 neurons; *N* = 4 mice)	0.047/*T* test	0.3
		V1 WT	V1 Lhx2 mutant	*p* value/test	*p* value (nested)
5	Total apical dendrite length (µm)	478 ± 23.8 (*n* = 38 neurons; *N* = 3 mice)	470 ± 34.9 (*n* = 30 neurons; *N* = 3 mice)	0.85/*T* test	0.92
6	Total basal dendrite length (µm)	570 ± 41.6 (*n* = 37 neurons; *N* = 3 mice)	587 ± 43 (*n* = 30 neurons; *N* = 3 mice)	0.77/*T* test	0.78
		V1 WT P30 (Swiss)	V1 Lhx2 OE (Swiss)	*p* value/test	*p* value (nested)
7	Total apical dendrite length (µm)	651 ± 45.5 (*n* = 23 neurons; *N* = 4 mice)	651 ± 64.5 (*n* = 18 neurons; *N* = 4 mice)	0.9950/*T* test	0.99
8	Total basal dendrite length (µm)	853 ± 36.2 (*n* = 23 neurons; *N* = 4 mice)	1,014 ± 45.6 (*n* = 21 neurons; *N* = 4 mice)	0.0085/*T* test	0.031

Values are given as mean ± SEM.

These findings indicate that LHX2 levels are lower in V1 progenitors than S1 progenitors and loss of *Lhx2* does not affect the dendritic arborization of V1 PyNs. Therefore, the role of LHX2 in regulating dendritic morphology is not uniform across these two sensory areas.

### *Lhx2* overexpression is sufficient to enhance the dendritic arborization of V1 PyNs

**Figure 4.**
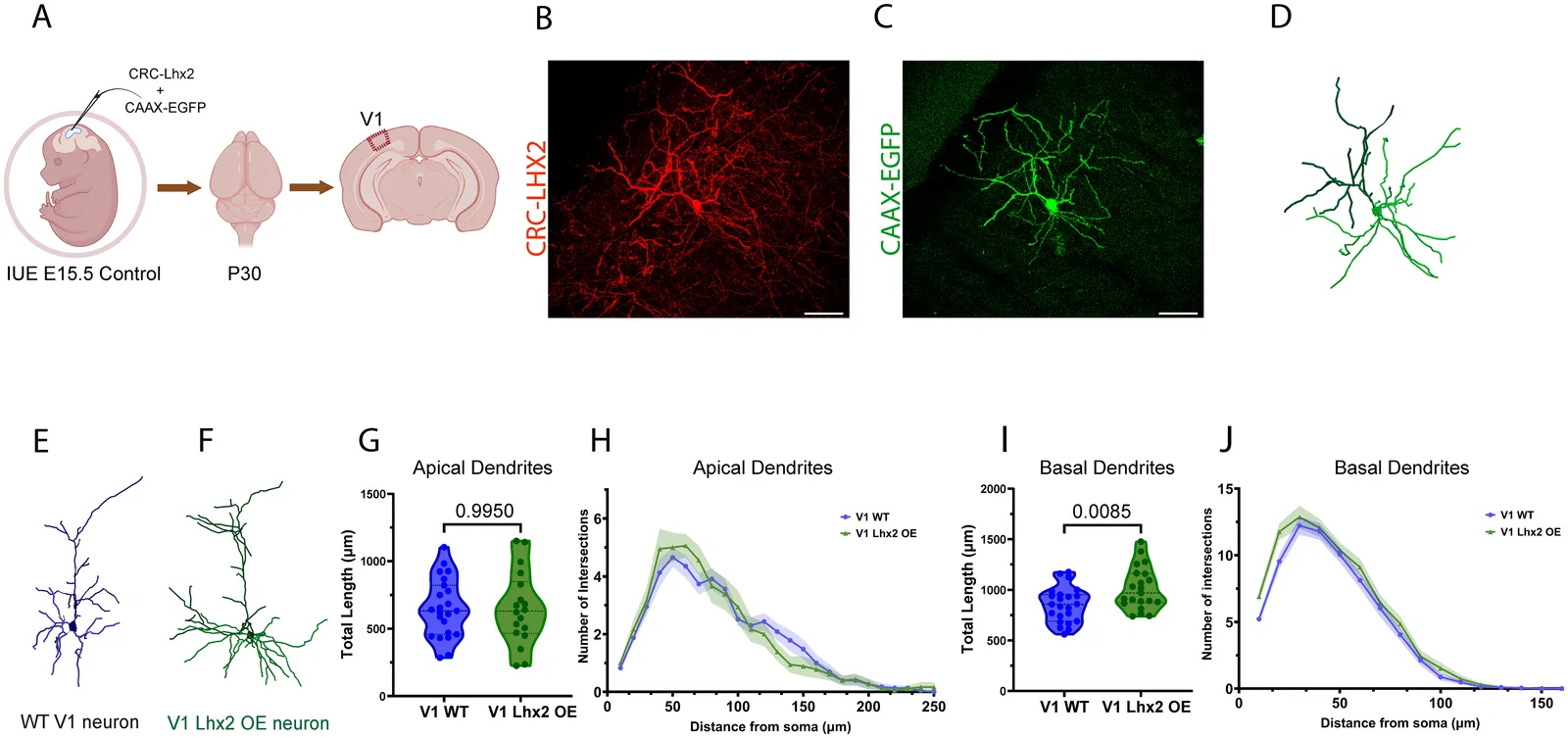
*Lhx2* overexpression is sufficient to enhance the dendritic arborization of V1 PyNs. ***A***, Schematic of *Lhx2* overexpression (OE) via IUE in V1. Swiss mice E15.5 embryos were electroporated with CRC-Lhx2 + CAAX-EGFP or just CAAX-EGFP to generate *Lhx2* overexpressing or control neurons respectively and analyzed at P30. ***B***, CRC-Lhx2 expressing P30 L2/3 V1 PyN (scale bar, 50 μM). ***C***, CAAX-EGFP labeled P30 L2/3 V1 PyN. ***D***, Dendritic morphology reconstruction of the neuron in ***B*** and ***C***. ***E***, ***F***, Representative dendritic morphology reconstruction of WT V1 and V1 Lhx2 OE neuron. ***G–J***, Morphological quantification, total apical [WT V1 = 651 ± 45.5 µm [*n* = 23 neurons; *N* = 4 mice]; V1 Lhx2 OE = 651 ± 64.5 µm [*n* = 18 neurons; *N* = 4 mice]; *p* = 0.99/0.99, *t* test/nested *t* test] and basal dendritic length [WT  V1= 853 ± 36.2 µm (*n* = 23 neurons; *N* = 4 mice); V1 Lhx2 OE = 1,014 ± 45.6 µm (*n* = 21 neurons; *N* = 4 mice); *p* = 0.0085/0.03, *t* test/nested *t* test] and Sholl analysis of WT V1 versus V1 Lhx2 OE neurons [blue V1 WT (wild type/control); dark green V1 Lhx2 OE]. Refer to Extended Data Figure 4-1 for nested comparisons of apical and basal dendrites between WT V1 and Lhx2 OE. A summary of all statistical comparisons is provided in Table 4.

We hypothesized that the lower levels of LHX2 in V1 progenitors may underlie the similarity in overall morphology between control V1 and *Lhx2* mutant S1 PyNs. To test whether augmentation of LHX2 levels enhances the dendritic arborization of V1 PyNs, we overexpressed a plasmid encoding *Lhx2* and tdTomato (pCRC-LHX2; see Materials and Methods) together with CAAX-EGFP in E15.5 control V1 progenitors using IUE and examined the resulting PyN dendritic morphology in juvenile mice (Fig. 4*A*–*D*). We found that *Lhx2* overexpression (Lhx2 OE) significantly increased the total basal dendritic length and proximal branching, while it did not affect apical dendritic morphology (Fig. 4*E*–*J*; Extended Data Fig. 4-1*A*,*B*; also see Table 4).

10.1523/ENEURO.0103-26.2026.f4-1 Figure 4-1 **Lhx2 overexpression is sufficient to enhance the dendritic arborization of V1 PyNs. A.** Nested Total dendritic length of V1 WT and V1 Lhx2 OE L2/3 PyNs of apical (V1 WT = 651 ± 45.5 µm, [n = 23 neurons, N = 4 mice]; V1 Lhx2 OE = 651 ± 64.5 µm [n = 18 neurons, N = 4 mice]; p = 0.99, Nested t-test) and **B.** basal dendrites (V1 WT = 853 ± 36.2 µm, [n = 23 neurons, N = 4 mice]; V1 Lhx2 OE = 1014 ± 45.6 µm [n = 21 neurons, N = 4 mice]; p = 0.031, Nested t-test). Download Figure 4-1, TIF file.

In summary, these data indicate that lower levels of LHX2 in V1 compared with S1 E15.5 progenitors may partially underlie the smaller dendritic morphology of the PyNs arising from these progenitors in V1 compared with S1.

## Discussion

In this study, we show that L2/3 PyNs exhibit area-specific structural and physiological differences across sensory cortices. Compared with neurons in primary somatosensory cortex (S1), PyNs in the primary visual cortex (V1) display smaller and less complex dendritic arbors. These structural differences were accompanied by distinct intrinsic membrane properties, with V1 neurons exhibiting higher IR, lower membrane capacitance, shorter membrane time constants, and increased intrinsic excitability compared with S1 neurons. Notably, these differences are already present by juvenile stages (∼P30), a developmental period that falls within the visual critical period, suggesting that areal specializations emerge before full maturation of visual cortical circuits rather than arising solely from prolonged sensory-driven refinement. Motivated by prior work from our laboratory showing that deletion of the transcription factor *Lhx2* in E15.5 S1 progenitors alters dendritic growth and neuronal excitability (Bose et al., 2025), we asked whether Lhx2 contributes to the differences observed between S1 and V1. We found that LHX2 levels are higher in progenitors of future S1 compared with V1. However, conditional deletion of *Lhx2* in V1 progenitors did not alter dendritic morphology, whereas increasing LHX2 levels modestly enhanced dendritic length and complexity. Together, these findings suggest that LHX2 contributes to shaping PyNs morphology in an area-dependent manner.

### Functional significance of morphology–excitability relationship

Consistent with the inverse relationship between dendritic complexity and intrinsic excitability, we find that this relationship also holds between L2/3 V1 and S1 PyNs (Eyal et al., 2014). In neurons with more compact dendritic arbors, reduced membrane surface area lowers capacitance and increases IR, effectively increasing neuronal gain. This structural–functional scaling allows smaller neurons to achieve higher firing rates for the same input current (Mainen and Sejnowski, 1996). Accordingly, V1 neurons exhibit lower rheobase and higher IR compared with S1 neurons. This structural–functional scaling suggests that V1 neurons may be tuned for high sensitivity, allowing them to reach firing threshold in response to sparser inputs (Douglas and Martin, 2004; Harris and Shepherd, 2015). Visual cortex processing places strong demands on temporal precision and the detection of rapidly changing sensory signals. Smaller dendritic arbors and higher IR may reduce electrotonic filtering, allow faster membrane charging, and enhance responsiveness to synaptic input, thereby supporting rapid gain control and efficient feature detection (Sangameswaran et al., 1996; Branco and Häusser, 2011). In contrast, the somatosensory cortex integrates convergent tactile inputs from multiple whiskers and incorporates contextual and motor-related signals during active sensing (Lefort et al., 2009). Larger dendritic arbors in S1 neurons may allow integration across broader synaptic inputs and provide the necessary surface area for segregating top–down motor signals from bottom–up sensory drive (Petreanu et al., 2009; Manita et al., 2015), while their lower intrinsic excitability may promote input selectivity and prevent excessive firing during sustained tactile exploration. Although these differences are already present by P30, suggesting that they emerge during early circuit assembly, sensory-driven and spontaneous activity likely still contribute to the maturation of these neurons and refinement of their synaptic inputs (Yang et al., 2018; Brandalise et al., 2025).

### Transcription factor levels as a mechanism for neuronal diversification

Transcription factors play a central role in regulating dendritic development, thereby contributing to the diversity of neuronal morphologies observed across the nervous system (Jan and Jan, 2010; Ledda and Paratcha, 2017; Lefebvre, 2021). In many cases the effects of transcription factors are not binary but depend on their expression levels and cellular contexts. Changes in transcription factor dosage can alter DNA-binding affinity, dimerization, or interactions with cofactors, thereby modifying transcriptional programs and downstream cellular outcomes (Noviello, 2025). In some contexts, differences in transcription factor concentration can even shift their function between transcriptional activation and repression (Dong and Guertin, 2026). Such dose-dependent regulation provides a mechanism by which developing systems can generate diverse neuronal phenotypes using a shared set of molecular regulators. A well-characterized example of this principle comes from studies of the transcription factor CUT in *Drosophila*, where graded levels of CUT determine the dendritic complexity of dendritic arborization neurons (Grueber et al., 2003). Similar mechanisms appear to operate in the mammalian cortex, where the CUT homologs CUX1 and CUX2 regulate dendritic branching and arborization of upper-layer PyNs (Cubelos et al., 2010, 2015; Li et al., 2010). L2/3 PyNs which are heterozygous mutants of SATB2 show simplified dendritic arborization (Finn et al., 2026). Consistent with this idea, LHX2 has been shown to regulate the expression of *Cux2* in upper cortical layers (Yang et al., 2020), suggesting that LHX2-dependent transcriptional programs may influence dendritic development through downstream regulators of neuronal arborization. In the present study, the differential LHX2 levels in S1 and V1 progenitors may explain the morphological and physiological differences observed between L2/3 PyNs across these areas and suggest that a threshold level is required to execute its regulatory functions. This is supported by our finding that V1 neurons that arise from progenitors with lower LHX2 levels have comparable morphologies to *Lhx2* mutant S1 neurons, and complementarily, overexpression of *Lhx2* in V1 progenitors results in postmitotic neurons with increased basal dendritic complexity.

A recent study in humans implicated *LHX2* haploinsufficiency as the cause of a range of neurodevelopmental disorders including autism, microcephaly, and intellectual disability (Schmid et al., 2023). Nineteen individuals from 18 families were identified that carried deletions that resulted in missense or likely gene-disrupting variants in *LHX2.* Further characterization revealed altered missense variants either impaired ability to bind essential cofactor LDB1 or displayed altered localization within the nucleus, and some were also impaired in their ability to transactivate a luciferase reporter. Together, these findings identified the importance of normal dosage of *LHX2* during neurodevelopment in humans (Schmid et al., 2023).

Together with our findings, these studies raise the possibility that Lhx2 function may be sensitive to dosage in different cellular contexts.

### Context-dependent roles of LHX2

LHX2 has pleiotropic roles in cortical development and in particular in neocortical progenitors. It maintains the progenitor pool by regulating the proliferation of neocortical progenitors (Chou and O’Leary, 2013). Loss of *Lhx2* in E11 progenitors results in the production of subplate neurons that display profound electrophysiological deficits, and the thalamocortical axons invade the cortex prematurely (Pal et al., 2021). Furthermore, L5 neurons expand at the expense of L6 (Muralidharan et al., 2017). Later, during gliogenesis, loss of *Lhx2* causes an increased proliferation of astrocytes selectively localized to the upper cortical layers (Iyer et al., 2025). In this context, our finding of the differential role of LHX2 in S1 versus V1 is particularly intriguing. Loss of *Lhx2* from E15.5 S1 progenitors produces L2/3 neurons with reduced, shrunken dendritic arbors (Bose et al., 2025) but causes no such change in the corresponding V1 neurons. This does not necessarily imply a simple dose–response relationship. Rather, endogenous LHX2 levels in V1 may be sufficient for the relevant developmental programs, or loss of Lhx2 may be buffered by area-specific transcriptional networks. In contrast, overexpression may exceed a functional threshold and engage additional transcriptional targets, cofactor interactions, or chromatin states that are less strongly recruited under physiological conditions. Thus, Lhx2 may contribute to dendritic development in an area-specific manner, with its effects depending not only on whether it is present or absent but also on its expression level and the chromatin state of the neuron. Consistent with such a scenario, interactors and downstream effectors of LHX2 are diverse. It interacts with the NuRD chromatin remodeling complex (Muralidharan et al., 2017); it regulates the expression of multiple genes in the Wnt and Hippo pathways and in the pathways that regulate axon guidance and pluripotency (Suresh et al., 2023), and of direct relevance to this study, it acts via suppression of transcription factor NEUROG2 to modulate the dendritic arbors of S1 L2/3 neurons (Bose et al., 2025). It is possible that overexpression of *Lhx2* in V1 progenitors may recruit a similar mechanism to enhance the dendritic complexity of the resulting neurons.

### Limitations and future directions

While these findings offer significant insights, several limitations exist. The analysis was restricted to L2/3 PyNs, leaving open the possibility that other projection classes follow different rules of specialization. Furthermore, while *Lhx2* manipulation clearly altered structure, the specific impact on synaptic and circuit-level function remains to be seen. The timing of this influence is also complex; because IUE affects both progenitors and postmitotic cells, the exact window when LHX2 shapes dendritic growth is difficult to pin down. Technical constraints in the recording and modeling processes must also be considered. The use of K-gluconate in voltage-clamp experiments may have introduced space-clamp limitations, potentially masking the full strength of inputs from distal dendrites. Finally, since this study focused on only two sensory cortices, it is yet to be determined if these developmental principles hold true for other sensory systems or higher-order areas.

The dendritic morphologies obtained by IUE were significantly shorter than the morphologies obtained by biocytin-filled neurons from acute slices. Possible reasons could be due to the thickness of patched slices (300 μm) versus electroporated sections (50 μm). The patched neurons had a more complete morphology because they were likely deep in the section, since the neurons near the surface are unhealthy and not suitable for recording or morphological reconstruction. The difference in the extent of the complete dendritic morphologies resulting from the two procedures is likely to be due to these reasons. A comparison of dendritic morphologies obtained from biocytin-filled neuron versus electroporated neuron from S1 is shown in Extended Data Figure 3-3, *A* and *B*.

Our results offer several avenues for future research. A primary question is whether the area-specific dosage of LHX2 interacts with specific extrinsic cues, such as thalamocortical input, to fine-tune neuronal identity. Future studies employing cell-type–specific recording could reveal if these morphological shifts translate into differences in sensory processing in vivo, such as receptive field size or orientation selectivity. Furthermore, exploring whether similar transcription factor gradients exist in higher-order association areas could uncover a universal molecular code for cortical diversification. Ultimately, bridging the gap between these genetic factors and functional circuit assembly will be essential to understanding how the neocortex achieves its functional diversity.

## Synthesis

Reviewing Editor: Matthew Grubb, King's College London Decisions are customarily a result of the Reviewing Editor and the peer reviewers coming together and discussing their recommendations until a consensus is reached. When revisions are invited, a fact-based synthesis statement explaining their decision and outlining what is needed to prepare a revision will be listed below. The following reviewer(s) agreed to reveal their identity: Kevin Bender.

Synthesis:

Both reviewers are positive about the study overall. They also identify issues which require resolution in a revised submission. Please directly address all of the Reviewers' comments as written below, with the exception of the following issues, for which we agreed an acceptable way forward in our consultation:

- we strongly urge the authors to include some images and analysis of Lhx2 expression in sagittal sections containing both S1 and V1 (see Reviewer 1, first point)

- on Reviewer 1's point regarding morphological measures from biocytin- versus GFP-labelled cells, we do not insist that all experiments are replicated with biocytin fills! But please explicitly acknowledge the differences in morphological values obtained from biocytin preps vs GFP label, and include a section in the Discussion that explores why these differences might exist (and whether or not they matter for the conclusions drawn from these experiments)

- Reviewer 1 suggested nested stats for the data in Fig3. On reflection &consultation, we agreed that a 2-way repeated-measures ANOVA (with one within-subject variable - apical/basal and one between-subjects variable - WT/manipulation) would be more appropriate to perform on these data.

- Both reviewers request that the data showing the effects of conditional deletion of Lhx2 in S1 should be included in this manuscript. To allow the direct comparisons on which the main message of this paper depends, this should take the form of S1 data from the same mice that were used for the V1 experiments shown in the current manuscript's figure 3. Statistical tests should also be used which allow for direct comparison of the effects of Lhx2 manipulation across cortical areas (e.g. 2-way ANOVA with cortical area and Lhx2 manipulation as main factors, explicitly testing for a significant interaction between these factors. This could be expanded to include within-subject apical/basal comparisons, as suggested above).

Reviewer 1:

This manuscript aims to detail how L2/3 cortical PyNs from different cortical regions have distinct morphological and excitability properties. Further, they argue that Lhx2 is a critical mediator for dendrite arborization in S1 but not V1 neurons. There is a clear arborization phenotype between V1 and S1 L2/3 neurons, along with some physiological differences that are largely descriptive in nature.

The authors show that Lhx2 deletion limits the dendritic arborization size of S1 L2.3 pyramidal cells, but has little to no effect on similar cells in V1. This is an interesting observation, potentially helping account for areal differences in dendritic complexity. The work could be improved considerably by further analysis, corroborative experimentation, and proper statistical approaches, as outlined below:

Fig 3A suggests that expression levels of Lhx2 are different between S1 and V1. This is shown with relatively high magnification images of progenitors in each region from coronal sections. This method makes it somewhat difficult to make direct comparisons between regions, as they were not stained within the same section. It should be possible to collect a saggital section containing both regions, which would allow one to determine if Lhx2 staining intensity gradually decreases from posterior to anterior cortical regions or is more abrupt as one moves into V1. This or some normalization approach to account for potential differences in staining quality across regions.

A second, independent approach to validate this one IHC observation would considerably strengthen the claim regarding Lhx2 levels. RNA scope and or western blot would be appropriate.

Extended data fig 1: There is a concern that some cells have had their dendritic arbors severed, limiting interpretability. S1, the 1st, 5th, 6th, and 8th cells have only either apical or basal dendrites. How are these cells considered in the analysis?

Related to this, the authors report similar differences between V1 and S1 in Lhx2 conditional deletions (Extended figure 5), the overall dendritic length is about 50% lower compared to WT juvenile mice from biocytin experiments. Is this difference due to approach (comparing biocytin vs tdTomato cell fills) or does Lhx2 conditional deletion impact dendritic arborization of both V1 and S1? One could imagine that biocytin filled cells could have been closer to the slice surface and have some processes severed at the slice surface. Therefore it is important to make comparable measures using the same approach. For example, patch as you did for WT biocytin fills.

Figure 3 is quite confusing. The legend states 3E is V1 Lhx2 mutant while label in the figure has it as S1 Lhx2 mutants. The manuscript text states it is S1 Lhx2. My guess is that it should V1 Lhx2 given the total length values for apical and basal dendrites in the extended data are nearly double for S1 compared to what is reported in Figure 3. Similar confusion is present for 3K. Please correct these issues.

Related to figure 3, a direct comparison of WT S1 to mutant S1 (even if this recapitulates prior figures) is useful context. And the authors should strongly consider proper statistical approaches. Right now it appears that the apical and basal dendrite lengths are considered to be independent measures, despite arising from the same cells. This approach requires nested statistical approaches.

Please specify the acquisition and Bessel filter rates in the Methods and state whether pipette capacitance was compensated (and to what degree) in current clamp. From the phase-plane plots, it appears that the rising phase of the AP waveform is filtered in some examples (a lack of distinct AIS and somatic components). This can be due to over-filtering, high series resistance, a lack of capacitance compensation, high pipette capacitance, etc. Regardless, since not much is made of peak dV/dt, it may be better to place phase plots in the supplement.

Extended data in Fig 4 appear to be analyzed on an event-by-event level rather than by cell. This can lead to spurious statistical significance. Synaptic data should be averaged per cell and statistics done using each cell's mean as the N.

Minor: Figures could be improved considerably. Color coding the apical dendrite the same in all examples (and the basal dendrites some other consistent color) may be useful. Data on violin plots could be clearer. Text is often very small.

Minor: Line 55, the last sentence of the paragraph does not flow clearly from the rest of the paragraph. Are the authors suggesting that all L2/3 neurons are generated at ~E15.5, independent of cortical region, and that this creates an issue when thinking about diversity in final arborization? The two do not necessarily follow each other. Perhaps this could be motivated differently.

Reviewer 2:

This study provides a detailed characterisation of fundamental differences in dendritic structure and intrinsic physiological properties between layer II/III neurons in V1 and S1 cortex. While previous work has relied on transcriptomic approaches to suggest differences between excitatory neurons across sensory areas within the same layers, this manuscript validates these distinctions at both the morphological and functional levels. Importantly, the authors demonstrate that these differences are not restricted to the adult brain but are already present at juvenile stages, when some of these neurons have not yet fully matured. Building on prior work identifying a role for the transcription factor Lhx2 in regulating dendritic growth in S1 neurons, the authors further show that Lhx2 can also modulate dendritic growth in V1 neurons, albeit in a more modest and context-specific manner under overexpression conditions.

Overall, I believe this manuscript is suitable for publication. However, substantial revisions are required before it is ready for publication.

General comments (Results + Discussion)

- Figure legends: For all figures, the number of cells, number of animals, statistical tests used, and significance levels should be clearly stated in the figure legends.

- Results (lines 262-264): The current phrasing "This analysis revealed that the apical dendritic compartment was significantly reduced in V1 compared to S1 neurons and the basal dendritic compartment showed a similar trend" does not fully reflect the results. The authors should clearly describe the findings regarding both apical and basal dendritic length and complexity, using more precise and explicit language.

- Results (lines 281-283; Extended Data Fig. 2H): When referring to the 3D plot, the authors should justify why these three specific properties were selected (this also applies to the equivalent analysis in juvenile mice). In addition, the claim that two clear populations are present ("The neurons from the two cortical areas occupied distinct regions of this space revealing their separability") is not convincingly supported by the current plot. The separation appears clearer in the juvenile dataset. As presented, the plots are difficult to interpret in terms of spatial dimensions, and it is challenging to distinguish the groups. I strongly recommend reconsidering the visualisation (e.g., improving clarity of axes, point distribution, and colour scheme). A clearer representation would greatly strengthen this point.

- Results (lines 304-312): The authors introduce the idea that structural differences between V1 and S1 may relate to differences in synaptic input. However, the conclusion of this section does not explicitly link the results back to this initial hypothesis. While differences in synaptic parameters are described, the interpretation should more directly address whether and how these findings support a relationship between dendritic structure and synaptic input properties.

- Results section: "Conditional deletion of Lhx2 affects dendritic morphology in S1 but not in V1 PyNs." Although the role of Lhx2 in shaping dendritic morphology in S1 neurons has been previously reported (Bose et al., 2025), the authors should still present the corresponding data within this experimental dataset. Including this comparison would both confirm prior findings and demonstrate reproducibility within the current study. I suggest including a direct comparison of S1 WT vs S1 mutant neurons in Extended Data Figure 5.

- The comparison between Lhx2 knockout and overexpression in V1 neurons is particularly interesting, as it suggests that while knockout has no detectable effect, overexpression produces a modest but measurable phenotype. While the Discussion addresses transcription factor levels as drivers of neuronal diversity, it does not explicitly consider a potential dose-dependent role of Lhx2 in V1 neurons. The authors should expand on this point and discuss possible mechanisms underlying the differential effects observed in knockout versus overexpression conditions.

Materials and Methods

Overall, the Methods section would benefit from additional detail. Specific points are outlined below:

- Image acquisition: More detailed information should be provided, including image resolution (e.g., image dimensions and pixel size). The authors should also clarify acquisition settings: for morphological analyses, were images acquired near saturation to maximise signal? For Lhx2 intensity quantification, it is essential that images are below saturation if intensity values are compared. Please specify these parameters and describe how intensity measurements were quantified.

- Whole-cell current-clamp recordings (cell identification): Please clarify how excitatory neurons were identified beyond anatomical criteria. Some interneurons can display pyramidal-like morphology. If any cells were excluded post hoc based on morphology and/or electrophysiological properties, this should be explicitly stated.

- Whole-cell current-clamp recordings (series resistance): The authors state that neurons were discarded if series resistance exceeded 30 MΩ. Was stability of series resistance also monitored throughout the recording (e.g., before and after protocols)? If so, please specify the criteria used.

- Statistics: In the statement "The data obtained was first assessed for normal distribution using normality tests", please specify which tests were used to assess normality.
